# Translation and psychometric evaluation of Self-Care of Hypertension Inventory Version 3.0 (SC-HI v3.0) in Iranian population

**DOI:** 10.3389/fpubh.2025.1423923

**Published:** 2025-03-26

**Authors:** Fatemehzahra Naddafi, Alireza Jafari, Elyas Hosseinzadeh Younesi, Moosa Sajjadi

**Affiliations:** ^1^Department of Geriatric Health, Faculty of Health, Tabriz University of Medical Sciences, Tabriz, Iran; ^2^Student Research Committee, Tabriz University of Medical Sciences, Tabriz, Iran; ^3^Department of Health Education and Health Promotion, School of Health, Social Development and Health Promotion Research Center, Gonabad University of Medical Sciences, Gonabad, Iran; ^4^School of Nursing, Faculty of Nursing, Nursing Research Center, Gonabad University of Medical Sciences, Gonabad, Iran; ^5^Department of Medical-Surgical Nursing, Faculty of Nursing, Nursing Research Center, Gonabad University of Medical Sciences, Gonabad, Iran

**Keywords:** psychometric, validity, self-care, high blood pressure, reliability, hypertension, Iran

## Abstract

**Introduction:**

Self-care is a key element of hypertension control. This study was carried out with the purpose of translating and investigating the psychometric features of the Persian version of Self-care of Hypertension Inventory Version 3.0 (SC-HI v3.0) in Iranian hypertensive population.

**Methods:**

This cross-sectional study was carried out in 593 people with hypertension in 2023 in Gonabad city (Iran). The participants were included by stratified random sampling from the community health centers of Gonabad city. The translation of the scale was done according to the multi-stage guideline of the instrument developers. The validity of the SC-HI v3.0 was examined by qualitative face validity, qualitative and quantitative content validity and construct validity assessed by confirmatory factor analysis (CFA). The reliability of the SC-HI v3.0 was also evaluated by calculating intraclass correlation coefficient (ICC) and Cronbach’s alpha coefficient. Data analysis was done using Amos software version 24 and SPSS software version 25.

**Results:**

In the translation process, face validity, and content validity evaluation, almost all the items of SC-HI v3.0 were partially revised without changing the main concept. In the construct validity evaluation, the results of CFA confirmed the Persian version of SC-HI v3.0 with 21 items and including three scales of maintenance with two factors (7 items), monitoring with one factor (7 items), and management with one factor (7 items). Finally, in reliability evaluation, Cronbach’s alpha coefficient and ICC were 0.879 and 0.842, respectively.

**Conclusion:**

Considering the lack of a comprehensive and brief tool for measuring hypertension self-care in Iran, the Persian version of the SC-HI v3.0 with 21 items and 3 scales, can be a valid and reliable inventory for self-care evaluation in Iranian hypertensive patients.

## Introduction

Hypertension (HTN) is a progressive cardiovascular syndrome that is usually defined as a chronic increase in systemic arterial blood pressure above a certain range (1). Today, HTN is a serious global threat for two reasons of high prevalence and dangerous complications. In the worldwide, 1.28 billion people (30–79 years) have HTN and also, HTN known as the silent killer, is one of the most prominent risk factors for coronary artery disease and stroke, and is one of the risk factors for kidney disease, heart failure, retinopathy, left ventricular hypertrophy, and peripheral arterial disease. As a result, HTN is considered the most important cause of disability and death all over the world (2–7). In Iran, statistics from 1990 to 2018 indicated an increase in the prevalence of this disease; so that one out of every four Iranians is suffering from HTN (8, 9).

The best way to control HTN is self-care (10). Self-care means the ability and implementation of adaptive actions of communities, families, and individuals in order to prevent diseases, restore or maintain health, adapt to disability or illness, and promote health (11, 12). Therefore, it is necessary to improve self-care in order to better control HTN and reduce the risk of stroke and heart diseases (13). Self-care in HTN includes activities such as medication adherence, blood pressure monitoring, low-fat and low-sodium diet, smoking cessation, alcohol restriction, weight loss, daily exercise, stress reduction, and regular health check-ups (14, 15). Lack of self-care in HTN is associated with increased hospitalization rates, increased risk of heart attacks and strokes (16).

The existence of a brief and also comprehensive instrument for self-care evaluation in HTN can facilitate the implementation of effective self-care interventions. In a systematic review, it was found that the current tools for evaluating the HTN self-care were not able to cover all the components of self-care and some of these tools, which were more comprehensive, had insufficient psychometric levels. For example, the Morisky tool and Hillbone tool only examine the adherence to the drug regimen and are not comprehensive (17). Also in 2014, a tool called Hypertension Self-Care Profile (HTN-SCP) was introduced by Han et al. This tool had 20 items and one-dimension and despite the coverage of different self-care behaviors in HTN, this tool has less addressed the monitoring and management aspects of self-care in HTN (18). In 2019, Eghbali-Babadi et al. (19) developed and evaluated the psychometric properties of the hypertension self-care questionnaire. This questionnaire had 25 items, but the monitoring and management aspects in HTN were less considered (19). Also, tools such as the Self-Care Inventory presented by Luciani et al. (20) was very general and measured self-care in general population, which cannot be used specifically in this patients.

Another instrument developed for self-care evaluation in hypertensive people is the Self-care of Hypertension Inventory (SC-HI). This inventory was developed by Dickson et al. (21). SC-HI is grounded in Self-care of Chronic Illness theory and includes 23 items and three scales of management, maintenance, and monitoring. Management in self-care means the responses and reactions that the patient shows when signs and symptoms appear. Maintenance in self-care means the implementation of behaviors and actions to maintain emotional and physical stability by a patient suffering from a chronic disease; and finally, monitoring in self-care means observing and investigating changes in signs and symptoms (21, 22). This inventory has been evaluated in different languages such as Arabic, Chinese, Turkish, etc. Also, this inventory measures different aspects of self-care compared to other tools; which shows the superiority and comprehensiveness of this inventory (10, 23, 24).

Due to the lack of a comprehensive and also brief instrument for measuring HTN self-care in Persian language, this study was performed with the aim of translating and psychometrically evaluation of SC-HI v3.0 in Iranian population.

## Methods

### Study design and participants

The present study is a cross-sectional study that was carried out from July to October 2023 with the aim of translating and evaluating the psychometric properties of SC-HI V3 in Gonabad, Iran. In our study, 593 hypertensive people were entered in the study by stratified random sampling. In Iran, all hypertensive patients are under the care of community health centers. At first, the community health centers of Gonabad city were considered as stratums. Then, according to the population covered by each center, sampling was done by simple random sampling. In Iran, the health records of people with HTN are available in the electronic system of community health centers. As a result, this electronic system was selected as the most comprehensive framework available for sampling hypertensive people. Then, the researcher visited the centers and, according to the number of samples calculated for each center, simple random sampling was performed. The selected individuals were contacted to visit the health centers if possible and, while performing routine checkups, the study process was explained to them by the researcher and, if they agreed, they completed the inventory in a self-report manner. If a person was unable to complete the questionnaire due to low literacy or vision problems, the inventory was completed by interviewing the participant by the researcher. In this study, clinical diagnosis of HTN, informed consent, and the absence of cognitive or communication disorders were considered as inclusion criteria and incomplete completion (less than 85%) of the questionnaire as exclusion criterium. To perform factor analysis, it is suggested to take between 3 and 20 samples for each questionnaire item (25). In this study, 20 samples were considered for each inventory item. Considering that the tool has 23 questions and considering 30% sample drop, the final sample size for confirmatory factor analysis was determined to be 593 hypertensive people.

### Instruments

#### Demographic part

This part was used to collect basic information and background characteristics of the participants and includes information such as sex, employment status, age, education level, income level, marital status and place of residence.

#### Self-care of Hypertension Inventory Version 3.0 (SC-HI v3.0)

This inventory was presented in English for the first time in 2017 and the third version in 2021 by Dickson et al. (21). SC-HI v3.0 is a reflection of Self-care of Chronic Illness theory and includes 23 items and three scales of Maintenance (9 items), monitoring (7 items) and management (7 items). This inventory also has a separate item (as a result, the inventory has a total of 24 items and the item number in the inventory is 17), which according to the developers, only the description of that item is sufficient and it is not a subset of these three scales (26). The items of this inventory have two types of five-choice Likert scale (never or rarely = 1, sometimes = with a three-choice spectrum of 2 or 3 or 4 and always or daily = 5 and not likely = 1, it is somewhat likely = with three-choice spectrum of 2 or 3 or 4 and is very likely = 5 and finally the last item with a six-option Likert scale, I did not do anything = 0, not sure = 1, somewhat sure = with three-choice spectrum 2 or 3 or 4 and very sure = 5). The score of each scale is between 0 and 100, and a higher score means better self-care (13, 21, 22).

#### Translation process

Translation of SC-HI V3 was done according to the instruction provided by the developers of the inventory. After obtaining the necessary permissions from the developers, first, the original SC-HI V3 in English was downloaded (from https://self-care-measures.com/) and used as the basis for translation. Then two translators independently translated the English version into Persian. As a result, in step 2, two versions of the “Persian language” were obtained, which were merged into one version in the third step. In the fourth step, two translators were used to back translate the merged Persian version into English. These two translators did the translation independently and were unaware of the original version. At the end of the fourth stage, two English versions were obtained, and in the fifth stage, the two English versions were merged and the last English version was obtained, which was sent and then confirmed by the main developer. Finally, the translated Persian version was also confirmed and final Persian version of SC-HI V3 was obtained (27).

#### Validity

The validity of SC-HI V3 was measured in three parts of face, content, and construct validity.

#### Face validity

Due to the standardization of SC-HI V3, in our study face validity was assessed only qualitatively. To assess qualitative face validity, SC-HI V3 was given to 10 hypertensive patients and 15 experts in nursing and health education and health promotion. Patients were asked to read each item and express their understanding of the items’ meaning. They were also asked to evaluate the items for appropriateness, level of difficulty and ambiguity. In case of any of the mentioned problems, the inventory was revised and the necessary corrections were made.

#### Content validity

The content validity of SC-HI V3 was evaluated first qualitatively and then quantitatively by 15 experts in nursing and health education and health promotion. To evaluate qualitative content validity, the items were evaluated for appropriate word application, grammar, simplicity, clarity, Likert scale and location of the items. If there was a problem in any of the mentioned areas, the items were revised and corrected. In order to evaluate the quantitative content validity, CVR (Content Validity Ratio) and CVI (Content Validity Index) were calculated. For CVR, each item was scored by experts using a 3-point Likert scale(it is necessary =3, useful but not necessary = 2, and not necessary = 1). To calculate CVI, each item was scored based on a 4-point Likert scale (completely It is related = 4, it is related = 3, it is related to some extent = 2 and it is not related = 1). In total, two indices of CVR, S-CVI/Ave were calculated for the whole instrument. The minimum acceptable value for CVR with 15 experts is calculated as 0.49 according to Lausche’s table (28). A CVI score more than 0.78 is desirable, between 0.79 and 0.7 is questionable and needs to be revised, and less than 0.7 is considered unacceptable and the item is removed (29). The average scores of the content validity index of the items (S-CVI/Average) is the index of the validity of the entire content of the questionnaire, and a score of 0.9 and above is considered favorable (30).

#### Construct validity

According to the developers’ basic articles (21, 22), this inventory consists of three distinct scales, with the first scale having two factors (autonomous and consultative behaviors) and the second and third scales each having a single factor. In this study, the first scale was considered with two factors, medical behaviors and lifestyle behaviors, and the second and third scales was also considered with one factor. The construct validity of SC-HI V3 was investigated using CFA. CFA was performed by AMOS software version 24. Before running CFA, the normality of the data was evaluated by checking the skewness and kurtosis. CFI, GFI, RFI, NFI, RMSEA, AGFI, IFI, χ2/df, PCFI and PNFI, were used to evaluate the final model. The acceptable rate of good fit indices are: χ2/df less than 5, PNFI more than 0.5, PCFI more than 0.5, CFI more than 0.9, AGFI more than 0.9, GFI more than 0.9 and RMSEA less than 0.08, IFI more than 0.9, RFI more 0.9 and NFI more than 0.9 (31–36).

#### Reliability

SC-HI V3 reliability was evaluated by calculating internal consistency reliability and stability reliability (test–retest). Cronbach’s alpha coefficient was calculated to evaluate the internal consistency reliability. Values of 7.0 and above are considered acceptable for Cronbach’s alpha (37, 38). To evaluate stability reliability, 30 participants completed the SC-HI V3 twice with an interval of 2 weeks, then the intraclass correlation coefficient (ICC) was calculated. The intra class correlation coefficient of 0.75 or more indicates proper stability (39). IBM SPSS software version 25 was used to calculate reliability coefficients.

## Results

### Demographic characteristics

In our study the response rate was 95% and 26 questionnaires were removed, due to incomplete information and after that 17 questionnaires due to outliers. Ultimately, data analysis was conducted on 567 participants. The mean of the age were 60.28 (SD = 12.91). In this study, 28.4% (*n* = 155) of participants were male and 71.6% (*n* = 391) of participants were female. Most of the participants were married (79.03%, *n* = 426), had elementary education level (32.3%, *n* = 172), were housewives (60.0%, *n* = 321), had medium income (78.02%, *n* = 387) and were city residents (74.45%, *n* = 373). Other demographic characteristics are described in more detail in Table 1.

**Table 1:** Frequency distribution of demographic characteristics (*n* = 550).

Variables		*n*	%
Sex	Male	155	28.4
	Female	391	71.6
Marital status	Married	426	79.04
	Single	6	1.11
	Divorced	6	1.11
	Death of spouse	101	18.74
Occupation	Housewife	321	60.0
	Employed	42	7.9
	Retired	121	22.6
	Self-employed	35	6.5
	laborer	16	3.0
Education level	Illiterate	93	17.5
	Elementary school	172	32.3
	Middle school	65	12.2
	High school	14	2.6
	Diploma	78	14.7
	Associate degree	29	5.5
	Bachelor degree	65	12.2
	Master’s degree or high degree	16	3
Economic status	Good	17	3.43
	Medium	387	78.02
	Weak	92	18.55
Place of residence	Village	118	23.55
	City	373	74.45
	Outskirts of city	10	2.0

### Face validity

In the phase of face validity using the qualitative method, items 1, 5, 10, 7, 11, 13, 14, 15, 16, 22, and 24 were revised slightly based on patients’ comments, without changing the main concept of the items.

### Content validity

Based on the expert panel, in the qualitative content validity, a number of items were revised slightly without changing the original concept. Also, according to expert panel, the expressions of the Likert scale were also modified based on the nature of the questions as follows; never (1), rarely (2), sometimes (3), most of the time (4) and always or daily (5) and unlikely (1), rarely (2), sometimes (3), most of the time (4) and it is very likely that (5). In quantitative content validity, the CVR of all items were greater than the calculated value (0.49) and none of the statements were deleted. Also, CVR and S-CVI/Ave were 0.950 and 0.987, respectively.

### Construct validity

**Figure 1:**
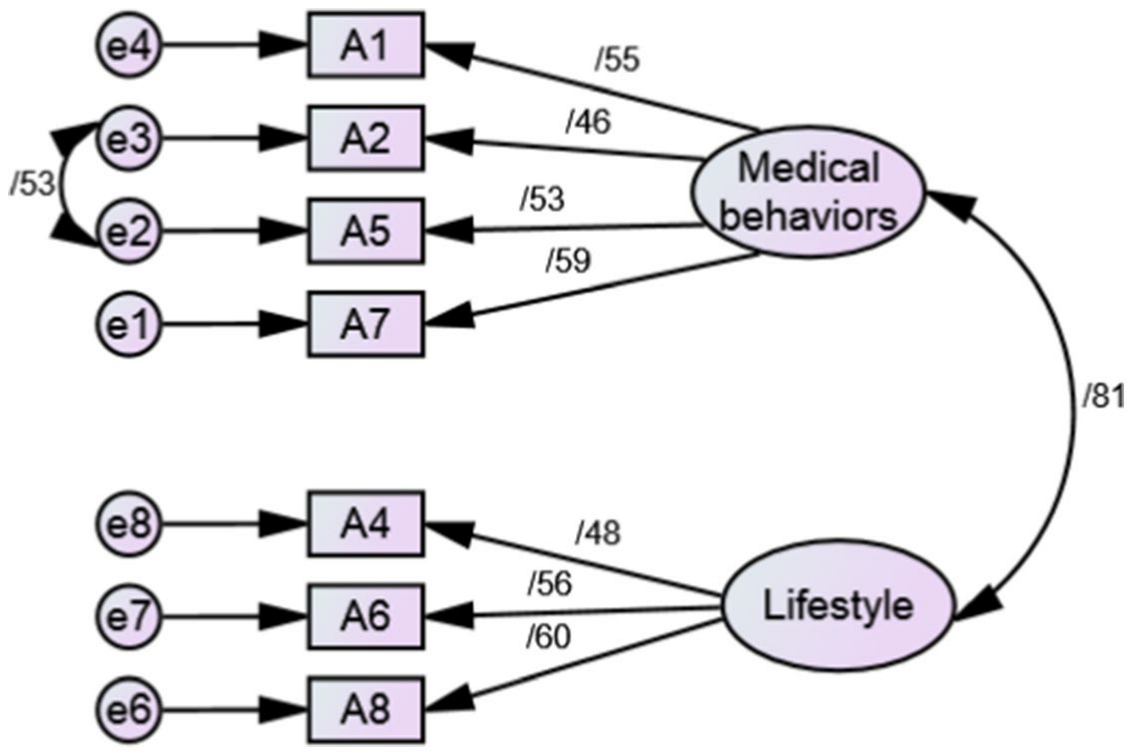
Standardized parameter estimates for the factor structure of maintenance scale.

**Figure 2:**
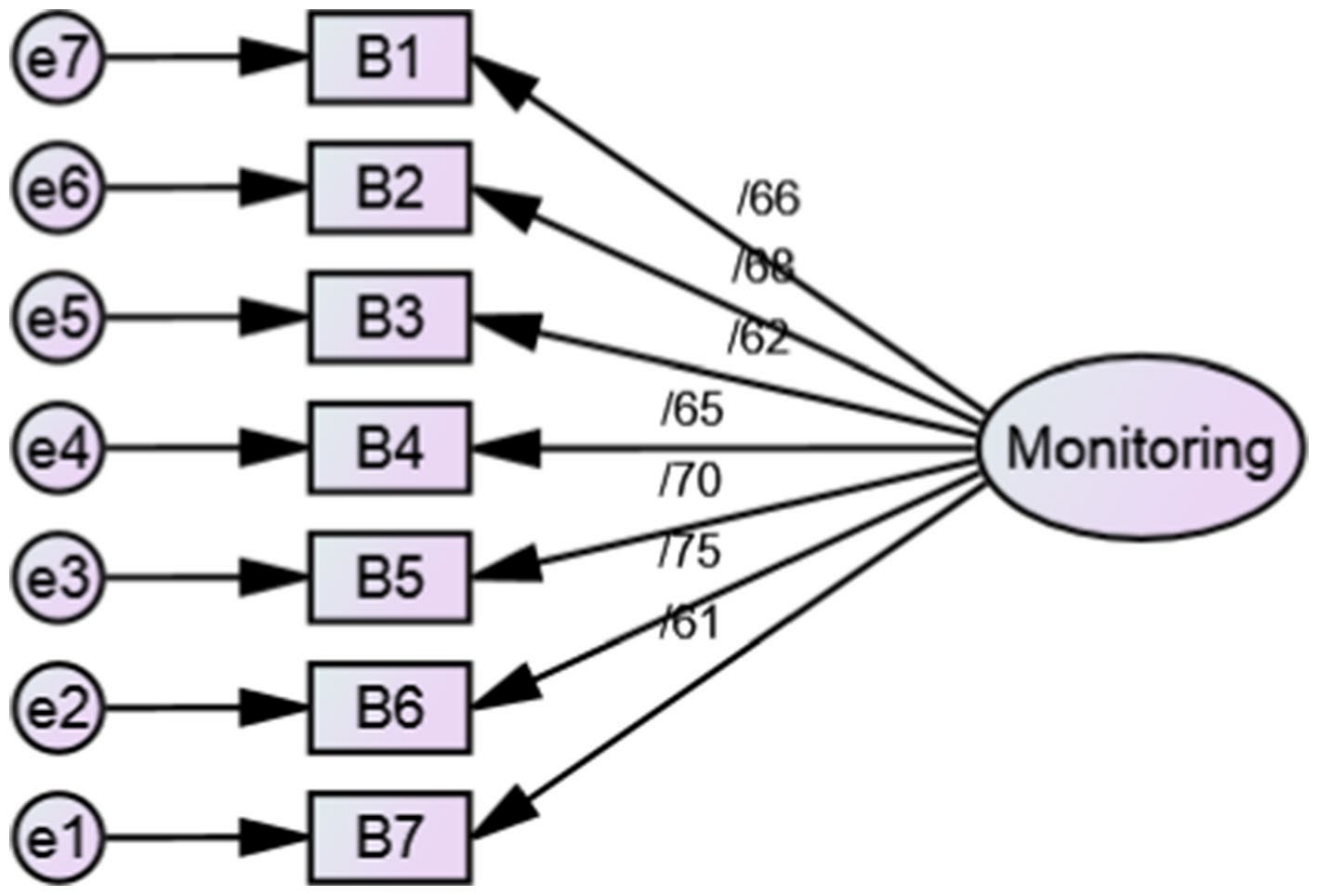
Standardized parameter estimates for the factor structure of monitoring scale.

**Figure 3:**
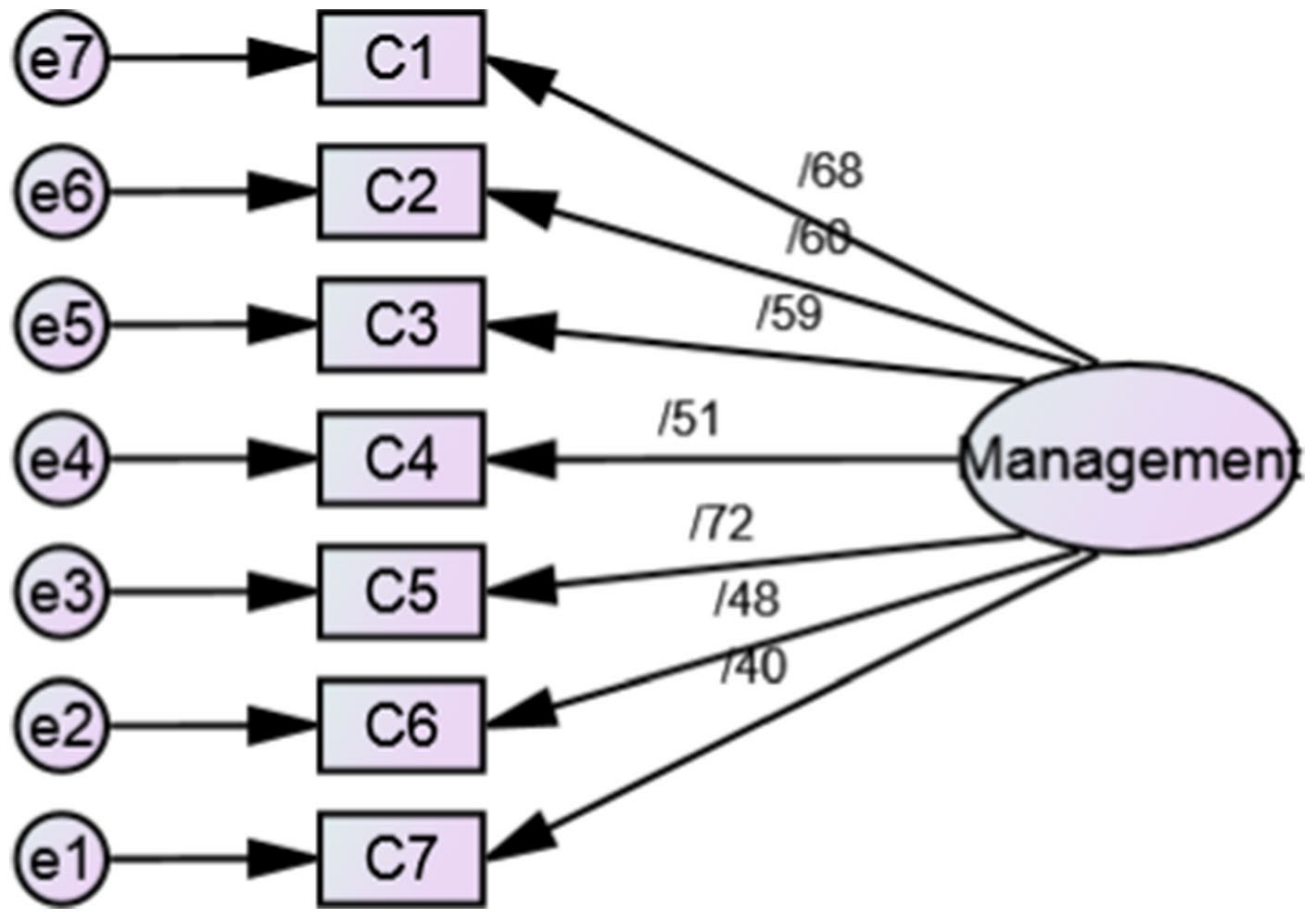
Standardized parameter estimates for the factor structure of management scale.

As a result, SC-HI V3 was evaluated with 23 items and three scales. Only the factor loading of two items (Table 2) was below 0.4, so these two items (item3 and item 9) were removed. Based on the results of goodness of fit indices, SC-HI V3 with 21 items and three scales of maintenance (7 items), monitoring (7 items), and management (7 items) was confirmed (Table 3, Figures 1–3).

**Table 2:** Factor loadings of Self-Care of Hypertension Inventory Version 3.0 (SC-HI v3.0).

Scales	Items	Factor loadings
Maintenance	1. Keep appointments with your healthcare provider?	0.552
	2. Take your blood pressure pills?	0.462
	3. Do something to relieve stress (e.g., medication, yoga, music)?	*Deleted*
	4. Do physical activity (e.g., take a brisk walk, use the stairs)?	0.484
	5. Take prescribed medicines without missing a dose?	0.526
	6. Ask for low salt items when eating out or visiting others?	0.562
	7. Try to avoid getting sick (e.g., flu shot, wash your hands)?	0.594
	8. Eat fruits and vegetables?	0.604
	9. Avoid cigarettes and/or smokers?	*Deleted*
Monitoring	10. Monitor your condition?	0.659
	11. Pay attention to changes in how you feel?	0.682
	12. Check your blood pressure?	0.616
	13. Monitor whether you tire more than usual doing normal activities?	0.653
	14. Monitor for medication side-effects?	0.698
	15. Monitor for symptoms?	0.752
	16. Monitor your weight?	0.611
Management	18. Reduce the salt in your diet	0.681
	19. Take your blood pressure medicine regularly	0.603
	20. Call your healthcare provider for guidance	0.590
	21. Reduce your stress level	0.513
	22. Talk to your healthcare provider about this at the next office visit	0.725
	23. Reduce your caffeine intake (coffee, cola, tea)	0.475
	24. How sure were you that the action you used helped?	0.403

**Table 3:** The model fit indicators of Self-care of Hypertension Inventory Version 3.0 (SC-HI v3.0).

Goodness of fit indices	Maintenance scale	Monitoring scale	Management scale	Acceptable value
χ^2^	48.183	73.643	65.577	-
df	12	14	14	-
X^2^/df	4.015	5.260	4.684	<5
*p*-value	0.000	0.000	0.000	*p* > 0.05
CFI	0.951	0.954	0.936	0.9 <
RFI	0.890	0.917	0.882	0.9 <
GFI	0.976	0.963	0.966	0.9 <
IFI	0.952	0.954	0.937	0.9 <
RMSEA	0.074	0.088	0.082	< 0.08
PNFI	0.535	0.630	0.614	> 0.5
AGFI	0.944	0.926	0.932	0.9 <
NFI	0.937	0.944	0.921	0.9 <
PCFI	0.544	0.636	0.624	> 0.5

### Reliability

In this study, Cronbach’s alpha coefficient was calculated 0.879 for the entire SC-HI V3. Also, ICC for the entire SC-HI V3 was calculated 0.842. Cronbach’s alpha and ICC coefficients of each of the scales are listed separately in Table 4.

**Table 4:** Reliability indices of Self-care of Hypertension Inventory Version 3.0 (SC-HI v3.0).

Scales	Item	Cronbach’s alpha coefficients	Intraclass correlation coefficient (ICC)	95% confidence interval		*P*-value
				Lower bound	Upper bound	
Maintenance	7	0.715	0.858	0.703	0.932	<0.001
Monitoring	7	0.846	0.799	0.558	0.906	<0.001
Management	7	0.755	0.756	0.475	0.885	<0.001
Total SC-HI v3.0	21	0.879	0.842	0.563	0.933	<0.001

## Discussion

Self-care is the key element of HTN control (10). The existence of a comprehensive, appropriate and brief tool to measure HTN self-care can facilitate interventions that promote self-care in hypertensive patients (17). As a result, this study was carried out with the aim of translating and examining the validity and reliability of the SC-HI V3 in Iranian hypertensive people. The results indicated that the Persian version of the SC-HI V3 had good validity and reliability.

In the phase of qualitative face and content validity, a number of items were partially revised according to the opinions of patients and expert panel without changing the main concept. Also, the expressions of the Likert scale of the instrument were changed to some extent according to the opinion of the experts. In quantitative content validity, CVR and S-CVI/Ave of the questionnaire were 0.950 and 0.987, respectively, which indicates the appropriate content validity of the Persian version of SC-HI V3. In the construct validity evaluation, according to CFA results, the SC-HI V3 with 21 items and three scales of maintenance (7 items), monitoring (7 items) and management (7 items) was confirmed with good fit indices. In total, the findings of our research confirmed the Self-care of Chronic Illness theory as the basis of this inventory. In the reliability evaluation, the internal consistency was measured with Cronbach’s alpha coefficient and stability reliability was measured by calculating the ICC. These coefficients for the whole inventory were 0.879 and 0.842, respectively, which indicated the appropriate reliability of the Persian version of SC-HI V3.

The results of our study were in line with the outcomes obtained in the examination of the psychometric properties of the English and Thai versions of SC-HI (13, 21, 22). Dickson et al. (21) developed and examined the psychometric features of the SC-HI V3 English version. The results indicated the two-factor structure of maintenance scale and unidimensional structure of monitoring and management scales of the third version of SC-HI. Also, the results showed that SC-HI V3, with the creation of a new monitoring scale, is a valid reflection of the Self-care of Chronic Illness theory and it measures the basic components of HTN self-care well.

Also, in the initial version of this inventory, which included three scales: maintenance, management, and confidence, the maintenance scale (CFI = 0.973, RMSEA = 0.085) and confidence (CFI = 0.979, RMSEA = 0.071) were confirmed with one dimension, and the management scale (CFI = 0.998, RMSEA = 0.07) was confirmed with two dimensions (22).

Suwanno et al. examined the validity and reliability of the second version of the SC-HI in Thailand. The secondary version of this inventory lacked the monitoring scale and instead had the confidence scale. In this version, the maintenance scale showed three factors structure, management scale two factors structure and confidence scale with one factor structure. The results of the confirmatory factor analysis indicated appropriate fit indices. The reliability results also indicated the acceptable reliability of maintenance scale (*α* = 0.68 and *ω* = 0.70) and confidence scale (*α* = 0.89 and *ω* = 0.90), but the reliability coefficients of management scale (α = 0.62 and *ω* = 0.65) were low in this study (13).

In examining the psychometric properties of the Hypertension Self-Care Profile in the Iranian population, the Cronbach’s alpha of the entire instrument was 0.865 (40), and in the Eghbali-Babadi et al. (19) study, the Cronbach’s alpha of the hypertension self-care questionnaire was 0.833, while in our study, the Cronbach’s alpha of the SC-HI v3 was higher and 0.879.

In our study, the first scale was maintenance, which was confirmed with 7 items. ICC and Cronbach’s alpha coefficient of this scale were 0.858 and 0.715, respectively. This scale measures 9 key behaviors necessary to maintain blood pressure within the appropriate range. These behaviors are: drug therapy (2 items), visiting health care provider, having regular physical activity, stress control, consuming low-salt foods even at parties, prevention of getting sick, consumption of fruits and vegetables, and avoiding smoking. This scale includes both behaviors related to lifestyle modification and adherence to treatment, which were gathered based on guidelines, published articles, and the American Heart Association (22). Also, these behaviors are consistent with the guidelines on HTN provided by the World Health Organization (WHO) and the Center for Disease Control (CDC) (41, 42). In this study, two items were removed from this scale due to low factor loading. This may be due to the participants not understanding the items properly, or, due to their old age and relatively low literacy, concepts such as stress management techniques were not known in our society.

Having a healthy lifestyle as the first step in the treatment of HTN can play a role as much as a HTN drug (43, 44). Also, preventing the need to drug therapy, reducing the need for drug therapy, improving drug effects and preventing cardiovascular diseases are some of the effects of implementing these behaviors (44–46). As a result, lifestyle modification behaviors along with therapeutic behaviors can maintain blood pressure in a normal range. The evaluation of these behaviors in hypertensive patients can be very helpful, which is provided by this scale.

The second scale in our study was monitoring, which was confirmed with 7 items. ICC and Cronbach’s alpha coefficient of this scale were 0.799 and 0.846, respectively. This scale measures behaviors such as monitoring blood pressure and weight, performing periodical checkups, checking signs and symptoms caused by increasing blood pressure, dangerous signs and symptoms, and checking side effects of drugs. The only way to check the effectiveness of drug therapy and lifestyle modifications is regular blood pressure monitoring. This regular monitoring can even help to adjust drug dosage, drug change and treatment decisions (47). Changes in weight are also related to changes in blood pressure in such a way that weight loss is associated with a decrease in systolic and diastolic blood pressure, while weight gain is associated with uncontrolled HTN. Therefore, weight loss is a key strategy in HTN management (48, 49). As a result, regular monitoring of weight can be helpful in these patients. Side effects of drugs are also one of the reasons for poor treatment compliance in hypertensive patients (50). Therefore, monitoring these side effects and informing the health care providers can help to change the drug regimen and reduce the side effects and thus improve treatment compliance. Finally, this scale can be very effective by evaluating monitoring actions in HTN.

The third scale of SC-HI V3 was management, which was confirmed with 7 items. ICC and Cronbach’s alpha coefficient of this scale were0.756 and 0.755, respectively. This scale obtained the lowest reliability coefficient compared to other scales, which is consistent with the results of studies conducted in Thailand, Poland, and Brazil (13, 16, 51). In this scale, the possible actions that patients take when blood pressure increases to control and manage it, are evaluated; such as reducing salt and caffeine consumption, reducing stress levels, contacting a health care provider, and the level of confidence of patients about the effectiveness of the actions taken during increased blood pressure. Actions such as relaxation cause a decrease in diastolic and systolic blood pressure (52). Also, the consumption of substances containing caffeine such as coffee can cause a rapid increase in blood pressure, which should be reduced when the blood pressure increases (53, 54). As a result, this scale is useful for evaluating the management power of patients with HTN.

This inventory can partially meet the need of researchers to have a short, valid and reliable inventory. In addition, health care providers can use this inventory to measure the level of self-care of patients with hypertension. Also, in the field of patient education, this inventory can be used in the initial assessment and final evaluation.

One of the limitations of this study was that it was conducted in only one city in Iran (Gonabad) which may limit external validity, so it is suggested that future studies further investigate the psychometric properties of SC-HI v3 in diverse settings. Another limitation of this study is the lack of assessment of divergent or discriminant validity, and it is suggested that researchers in other regions of Iran further investigate these indicators. In Iran, people who are diagnosed with hypertension by a doctor often immediately join online health center systems, so it can be said that there is a very small percentage of people who may have been underrepresented. But, future studies should consider this issue. Among the strengths of this study compared to previous studies, we can mention the large sample size and the calculation of the external reliability index, i.e., intraclass correlation coefficient (ICC). Also, in the current study, face validity was assessed qualitatively and content validity was examined both qualitatively and quantitatively; that in previous studies, the evaluation of this validities has been given less attention.

## Conclusion

The Persian version of SC-HI V3 with 3 scales and 21 items had good validity and reliability. As a reflection of the Self-care of Chronic Illness theory, this inventory is able to evaluate essential components in HTN self-care. This tool is not only useful for measuring the level of self-care of hypertensive patients, but it can also be used to evaluate the effectiveness of lifestyle educational interventions and treatment compliance programs. It is also possible to use the scales of this inventory to check the level of monitoring and management ability of patients when blood pressure increases.

## Data Availability

The datasets presented in this study can be found in online repositories. The names of the repository/repositories and accession number(s) can be found in the article/supplementary material.
